# The Effect of Neurohormonal Factors, Epigenetic Factors, and Gut Microbiota on Risk of Obesity

**Published:** 2009-06-15

**Authors:** Matthew A. Haemer, Terry T. Huang, Stephen R. Daniels

**Affiliations:** University of Colorado School of Medicine, Pediatrics Section of Nutrition, The Children’s Hospital; Obesity Research Strategic Core, Eunice Kennedy Shriver National Institute of Child Health and Human Development, National Institutes of Health, Bethesda, Maryland; Daniels, University of Colorado School of Medicine and The Children’s Hospital, Denver, Colorado

## Abstract

Molecular, cellular, and epidemiologic findings suggest that neurohormonal, epigenetic, and microbiologic mechanisms may influence risk for obesity by interacting with socioenvironmental factors. Homeostatic and nonhomeostatic neural controls of energy predispose people to obesity, and this predisposition may be exaggerated by the influence of media, marketing, and sleep patterns. Epigenetic gene regulation may account for the influence of modifiable early life or maternal exposures on obesity risk. Alterations in gut flora caused by infant feeding practices or later diet may influence the absorption and storage of energy. Further exploration of how these molecular-cellular mechanisms might increase obesity risk in response to modifiable socioeconomic factors requires the partnership of laboratory and public health researchers.

## Introduction

We describe components of a novel paradigm for obesity-related research and public health interventions that are designed to generate cross-disciplinary hypotheses that account for multiple levels of causation from molecular to societal. The goals of this approach are to understand the causal pathways leading to patterns of obesity in populations and to identify where interventions that can broadly affect the population can be implemented (1). Our focus is on the mechanisms that may be useful for understanding how socioenvironmental factors interact with biological processes to affect energy balance. We review neurohormonal controls, epigenetics, and microbiologic mechanisms in gut flora that may influence risk for obesity.

## Neurohormonal Controls

### Homeostatic controls

The complex neurohormonal systems controlling weight and adiposity can be categorized as either homeostatic or nonhomeostatic (2). Parsimonious homeostatic mechanisms provided a survival advantage to people faced with periodic starvation. Organisms that could consume, store, and conserve energy efficiently were likely to survive and reproduce (3). Although evolution may have selected against extreme adiposity (4), the negative feedback signals against excess intake are insufficient to maintain normal body weight for most humans who have easy access to palatable, calorie-dense foods (5).

When provided a diet high in calories, animals prone to obesity rapidly increase fat stores (2). Furthermore, when obese rats lose weight or have their calorie intake restricted, they mount the same neurohormonal drive as do lean rats to increase intake and decrease energy expenditure, effectively defending obesity (6,7). After weight loss, the average resting energy expenditure of obese people is markedly and persistently reduced (8). These factors are blamed for the weight regain that occurs in approximately 80% to 90% of obese people who have lost weight (8,9).

A complex interplay of neurotransmitters, hormones, and metabolites regulates food intake in the brain. Metabolic sensing neurons of the hypothalamus and other brain areas respond to signals of energy intake, demand, or storage including circulating glucose, leptin from adipocytes, insulin, ghrelin from the stomach, adrenal steroids, polypeptide YY from the intestines, fatty acids, ketones, lactate, vagal nerve afferents, and intrinsic neurotransmitters (5). Hypothalamic neurons release neurotransmitters that activate either catabolic processes (eg, a-melanocyte-stimulating hormone [a-MSH], cocaine- and amphetamine-regulated transcript [CART], corticotropin-releasing hormone [CRH]) or anabolic processes (eg, neuropeptide Y [NPY], agouti-related protein [AGRP], orexins) (10). Hypothalamic neurons signal broadly to the pituitary, brainstem, midbrain, and forebrain. These pathways regulating energy intake and expenditure have been demonstrated experimentally in animal models and through functional neuroimaging in humans (11).

The interplay between the sympathetic nervous system and leptin signaling is an example of neurohormonal protection of adiposity. With calorie restriction, sympathetic activation releases glucose from glycogen stores and fatty acids from adipose tissue. Adipose tissue responds to sympathetic activity by markedly decreasing leptin production, which decreases resting energy expenditure and increases appetite to replenish fat stores (2). Some rare human obesity syndromes are associated with single gene defects within homeostatic pathways, including leptin deficiency, leptin receptor defect, pro-opiomelanocortin (POMC) deficit leading to impaired a-MSH production, and a-MSH receptor defects (12).

### Nonhomeostatic controls

Nonhomeostatic systems promote excess weight gain through responses to the reward properties of food and psychosocial factors associated with eating (5). When a palatable and calorically dense diet is provided, rats eat far beyond limits of homeostasis and develop extreme levels of obesity, even rats predisposed to leanness (13). The more palatable the diet, the higher the degree of obesity and the longer it is sustained (13).

The organization and function of the human brain is a reflection that throughout all but the most recent evolutionary history, obtaining food was a difficult task. Complex pathways record past context and the expectation of reward (14). Reward properties of foods (the stimuli that augment the drive to obtain foods) mediate "liking" by action at opioid receptors and "wanting" by action at dopaminergic receptors. Both of these receptors also mediate addiction (4). Metabolic signals modify the sensing thresholds for food-related stimuli, food-seeking behavior, and reward signals (4). Chronic stress enhances the reward value of foods (15). Although the subcortical areas contribute to subconscious drives for intake, the cortical areas integrate these underlying signals with learned motivational cues. These cues can drive intake well beyond subcortical demands of energy needs (16). Overall, the drive to eat is the result of complex, redundant systems that protect against starvation, but the systems are grossly mismatched to the food and activity environment of the developed world (17).

### Implications of neurohormonal mechanisms for obesity and public health

The potency of homeostatic and nonhomeostatic forces that promote weight gain and prevent weight loss make clear the value of obesity prevention, especially considering the cost and difficulty of obesity treatment. Many socioenvironmental factors interact with the neurologic drivers of intake, so identifying and intervening on modifiable levels to prevent obesity is a challenging task.

Sleep may play a role in maintaining proper energy balance by influencing neurohormonal controls (18). A meta-analysis of 30 cross-sectional studies through early 2008 found an odds ratio for obesity of 1.89 in children with short duration of sleep and 1.55 in adults with short duration of sleep (19). More recently published longitudinal studies, one with 32 years of follow-up from birth, also found that shorter sleep times in childhood were significantly associated with increased body mass index (20,21). Experimental studies of sleep deprivation show increased hunger and appetite associated with neurohormonal mechanisms that promote intake: decreased levels of leptin, increased levels of ghrelin, increased sympathetic tone, and increased cortisol (22). Nonhormonal effects of short sleep, such as fatigue and decreased volitional energy expenditure, may also play a role in the association between sleep and obesity. Debate remains about the strength of the evidence that poor sleep causes obesity, and interventions to decrease obesity by increasing sleep have yet to be reported (23).

Media exposure and food marketing either overpower homeostatic negative feedback or strongly amplify nonhomeostatic drivers of intake. Distracting stimuli, such as television viewing while eating, strongly increase intake, possibly by interfering with neural signals of satiety (24). In a controlled experiment, viewing children's food advertisements caused children to eat much larger portions of snack foods compared to children who watched nonfood advertisements, and the effect was significantly larger on obese children than on normal-weight children (25). Marketing often seeks to influence the emotional responses to food (26) and succeeds in altering the perceived reward value of foods (27). Most food advertisements targeting preschool children involve fast-food restaurants or sweetened cereals. These advertisements associate the products with fun and happiness in an attempt to create long-term customers through positive emotional associations with the product (28). The food industry targets children at stages of development critical to establishing future eating habits (28).

Analysis by the National Bureau of Economic Research estimated that eliminating fast-food restaurant advertising to children would reduce the prevalence of obesity by 18% (29). Some governments have restricted television advertising of food products to children, and some advertisers have voluntarily restricted advertisements (30). However, the recent proliferation of other digital media sources, including cell phones, mobile music devices, broadband video, instant messaging, videogames, and virtual worlds, has created a "marketing ecosystem" for advertisement of food (31,32), and the influence of marketing on intake may become more pervasive in the future.

## Epigenetics

Studies searching for determinants of risks for obesity and cardiovascular disease have found that genetics (33,34) and behavioral exposures (35) explain only part of the risk. Epigenetic mechanisms describe environment-gene interactions that may explain some residual risk. The term epigenetics refers to cellular mechanisms that affect gene expression without changing DNA sequence (36). Epigenetic markings can be inherited and modified throughout the lifespan (37). Epigenetic modifications during critical early periods, such as embryogenesis (38) have the most effect on phenotype. Fetal and early life exposures have been associated with numerous health outcomes later in life, including obesity (39,40). Changes to DNA marking and packaging may explain the influence of the environment on gene expression throughout a person's life and even across generations (41). Evidence is mounting from experimental studies in animals and from human epidemiologic studies that epigenetic mechanisms may affect risk for chronic disease, especially when the environment predicted by fetal experience does not match the environment later in life (42,43).

### Animal models of epigenetics related to obesity

Experimental studies have subjected animals to dietary, chemical, and stress exposures during prenatal or early life that lead to epigenetic changes in gene expression in adulthood. The epigenetic changes caused by some exposures are preventable by other agents. Some changes in gene expression persist across generations when epigenetic markings are incompletely erased during formation of the sperm and ova (38). Several animal studies illustrate epigenetic influences on obesity.

The *Agouti* (*A^vy^*/*a*) mouse is a well-described model of epigenetically controlled obesity. Among mice carrying 1 obese allele (*A^vy^*) and 1 nonobese allele (*a*), those with inadequate methylation of the *A^vy^* allele develop obesity and yellow fur (38). The *A^vy^* protein blocks satiety signals from insulin and leptin on the hypothalamus (37). Obesity is amplified through multiple generations of *A^vy^/a* mice, but supplementing the diet with methyl donors prevents this amplification (44). Maternal ingestion of bisphenol A (BPA), a chemical used in polycarbonate plastic and epoxy resins, decreased methylation of the *A^vy^* allele in offspring (45). This decrease in methylation did not occur when the soy isoflavone genistein or a methyl donor, such as folic acid or vitamin B_12_, was added to the BPA-containing diet (45).

Rats whose mothers do not eat enough protein during pregnancy have decreased methylation and have increased expression of glucocorticoid receptors and peroxisomal proliferator-activated receptor-a in the liver. These changes are associated with components of metabolic syndrome including hypertension, dyslipidemia, and insulin resistance. Giving mothers folic acid supplements prevented the hypomethylation and normalized the gene expression in offspring (46).

Elevated reactivity to psychological stress has been associated with obesity in humans (47). In a model of early life stress, rats who are poorly nurtured by their mothers develop exaggerated stress responses and poorly nurture their own offspring; the pattern is repeated through multiple generations (48). Poor nurturing increases stress reactivity through methylation and de-acetylation of the glucocorticoid receptor gene in the brain, decreasing receptor production (49). Infusion of histone deacetylase inhibitors or "adopting out" offspring to highly nurturing mothers prevented these changes (48). Epigenetic recording of early parenting interactions affects the phenotype throughout the lifespan in this model (50).

### Human evidence supporting epigenetic mechanisms of obesity

Certain genes may be particularly vulnerable to epigenetic changes. Metastable epialleles like the *A^vy^* allele in mice are subject to dramatic interindividual differences in methylation (37). Imprinted genes, for which the allele from 1 parent is normally silenced by methylation, are at risk of causing functional problems if the remaining copy is inappropriately silenced. Two rare human obesity syndromes, Prader-Willi and Beckwith-Wiedemann, can result from inappropriate methylation of imprinted genes (38). The search for metastable epialleles and imprinted genes affecting obesity in humans is of great interest.

Several studies have examined the effects of specific exposures on human fetuses or infants. A prospective study following 1,100 mother-child pairs from the prenatal period examined the effect of several perinatal exposures on obesity at age 3 years: prenatal smoking, excess weight gain during pregnancy, breast-feeding for fewer than 12 months, and poor infant sleep duration (51). This study found a progressive increase in risk of obesity with the addition of each risk factor (51). A meta-analysis of breast-feeding studies found a dose-response effect of breast-feeding duration on decreasing obesity prevalence. Each month of breast-feeding up to 9 months decreased the risk of obesity at 3 years by 4%, yielding an odds ratio of 0.68 for overweight in those breast-fed longer than 9 months compared with infants who were fed formula exclusively (52). These human studies did not include analysis of specific epigenetic markers for gene expression, but they suggest that early life exposures can have lasting effects on phenotype.

One study has demonstrated epigenetic changes in human growth-controlling genes associated with adverse perinatal events. The Dutch famine of 1944-1945 provides a unique opportunity to study humans exposed to well-defined undernutrition. Adults who were exposed to poor nutrition in utero had an increased prevalence of glucose intolerance, dyslipidemia, early coronary heart disease, and obesity (53,54). A recent study of adults who were exposed to the Dutch famine early in gestational development is reportedly the first to provide empiric support for the hypothesis that environmental exposures can cause epigenetic changes in humans. The insulin-like growth factor-2 (IGF-2) gene, which exerts control over fetal growth, is imprinted with the maternal allele normally silenced by methylation (55). Methylation of IGF-2 was decreased in adults who were exposed to famine early in utero compared with unaffected siblings and those exposed to famine only late in gestation. This was possibly an effect of decreased availability of methyl donors such as folate and the amino acid methionine during early development (43).

### Implications of epigenetic mechanisms for obesity and public health

If epigenetic modifications that increase risk for obesity and chronic diseases occur widely in humans, the implications for public health interventions could be substantial. Epigenetic changes could underlie the increased risk of central obesity and cardiovascular disease among adults who experienced adverse in utero conditions, but at present direct evidence in humans is sparse. Epigenetic modifications have been proposed as contributors to variation in obesity across race, country, and immigrant status (35,56). Some prenatal and early life exposures such as smoking, inappropriate weight gain during pregnancy, gestational diabetes, and early feeding are known targets for intervention, but a modifiable biologic mechanism may provide an additional incentive to intervene early. Maternal stress or chemical exposures may be explored for epigenetic mechanisms affecting human disease. Research related to epigenetics draws on the expertise of multiple disciplines, including molecular biology/genetics, nutrition, environmental epidemiology, and life course epidemiology. An international scientific initiative, the Alliance for the Human Epigenome and Disease (AHEAD), has a goal to create a reference map of epigenetic modification sites (57). Prospective studies that collect biological samples early in life, such as the National Children's Study, may provide insight into epigenetic mechanisms of obesity and chronic disease (58).

## Gut Microbiota

Differences in intestinal flora may explain some of the risk for obesity, yet like epigenetic mechanisms, evidence in humans is sparse. The gut microbiota consists of the microorganisms, predominantly bacteria, that inhabit the gastrointestinal tract. These microbes metabolize otherwise indigestible components of the diet, and the products of microbial metabolism affect the amount of energy absorbed (59). The combined gene pool of gut microorganisms enables absorption of simple sugars from complex polysaccharides and may influence fat storage by modifying lipoprotein lipase activity (60). RNA sequencing has been used to study bacterial species in stool samples (61). The species-level makeup of gut flora varies from person to person, but most analyses related to obesity have focused on how the relative proportion of 2 major bacterial divisions, Bacteroidetes and Firmicutes, differ between obese and lean people (62).

### Animal studies of microbiota and obesity

The effect of microbiota on energy absorption and obesity has been demonstrated in mice. Germ-free mice have significantly less body fat than do mice with standard microbiota, and rapidly gain weight after inoculation (60). Obesity-prone mice, in contrast to their nonobese siblings, carry microbiota with an enhanced proportion of genes to break down polysaccharides, and their stools show increased fermentation products and decreased residual calories (59). When gut flora from obese or lean mice were transplanted to germ-free mice eating a high-fat, high-sugar diet, those receiving microbiota from obese mice gained significantly more fat than did those receiving lean mouse flora (59).

### Human studies of microbiota and obesity

Thus far, human studies regarding the effect of intestinal flora have been observational and have had small sample sizes. A recent case-control study found gut flora in infancy predicted overweight later in childhood (63). Among children who were enrolled as newborns, 25 overweight 7-year-olds were matched with normal-weight controls for multiple factors including probiotics supplementation, antibiotic use, and breast-feeding duration (63). On average, overweight children had lower numbers of the genus *Bifidobacterium* spp. and higher numbers of *Staphylococcus aureus* in their stools during infancy (63). Bifidobacteria are the predominant flora of breast-fed infants and are hypothesized to affect weight gain through mucosal host-microbe crosstalk, immune regulation, and inflammation (64).

Other studies have examined the flora of obese adults. Studies of lean and obese twin pairs found a large variability among species of bacteria present, but a core group of functional genes was present across participants regardless of species type (65). In addition to this "core microbiome," the microbiomes of obese twins contained more genes involved in carbohydrate, lipid, and amino acid metabolism (65). A small study followed stool microbiota in 12 obese people randomized to low-fat or low-carbohydrate diets for a year. Obese participants had a smaller proportion of Bacteroidetes and a higher proportion of Firmicutes at baseline. During the study, the proportion of Bacteroidetes steadily increased as weight decreased on both diets, especially on the low-carbohydrate diet (66).

### Potential implications of gut microbiota for obesity and public health

Other reviewers have suggested that from a public health perspective, it might be wise to avoid shifting too much focus from the known modifiable causes of obesity to gut flora (62). With that reasonable caveat, a small constant source of increased energy absorption could have a substantial effect on obesity in populations.

Throughout history, many cultures have used beneficial microorganisms to create fermented foods containing live microorganisms capable of modifying the makeup of intestinal flora, such as yogurt, curd, and kefir (62). Wide-ranging health claims have been made of commercially available probiotics (live nonpathogenic organisms) and prebiotics (fermentable substrates that enrich the gut for potentially beneficial organisms). Larger prospective studies are required to validate these health claims and to evaluate whether experimental changes to gut flora can affect obesity (67). The genetic sequencing techniques necessary to carry out this type of research are rapidly evolving (68). The National Institutes of Health has devoted substantial resources to the Human Microbiome Project to study the association of microbiota from the gut and other body sites with disease processes (69).

## Conclusion

A systems approach to the obesity problem necessitates research that connects socioenvironmental factors with biological processes related to energy metabolism. By understanding how obesity results from the interaction of cellular factors with social factors, we can develop interventions that include molecular medicine and broad social policy. The potential biological drivers of obesity include evolutionarily conserved neurobiological mechanisms, epigenetic gene-environment interactions, and gut microbiota. These examples show the need for partnership among investigators across the spectrum of science. Research questions and hypotheses that are cross-disciplinary can aid development of interventions to prevent or control obesity at multiple levels.
